# Parametric contribution of building form to whole-life carbon decision-making

**DOI:** 10.1098/rsos.241953

**Published:** 2025-07-16

**Authors:** Danielle Abbey, Hadi Arbabi, Danielle Densley Tingley

**Affiliations:** ^1^ School of Mechanical, Aerospace and Civil Engineering, The University of Sheffield, Sheffield, UK

**Keywords:** retrofit, building form, whole-life carbon, sustainable buildings

## Abstract

Existing buildings generate 30% of global emissions because of the energy required to heat, cool and power them. Mass improvements in building fabric efficiency and heating/cooling systems are therefore imperative. Fast-running modelling approaches are thus necessary to identify appropriate interventions for the global building stock. This paper presents a new parametric formulation to determine the best whole-life carbon intervention as a function of building form. We demonstrate that buildings of inefficient form have greater potential for energy savings, providing a useful prioritization tool for future planning decisions. We present results as a novel graphical tool, which can be used to identify the lowest carbon scenario for any building form across a combination of building storeys and glazing ratios. This is applied to a cool-temperate climate, comparing a retrofit scenario, to the option of replacement with new construction. Finally, we apply the formulation to a subset of the UK educational building stock, assessing 15 193 forms. For this scenario, we conclude that retrofit always results in lower whole-life carbon compared to replacement with attainable new construction standards. This work provides practical assistance with early stage decision-making and theoretical understanding of how form influences energy consumption and whole- life carbon emissions.

## 1. Introduction

Global building stocks account for approximately 40% of total carbon emissions, with 70% from operational energy consumption and 30% from the embodied impacts of materials [1]. Carbon mitigation is urgently needed in the built environment sector to reduce the future impacts of climate change. In cold and cold-temperate climates, current and future operational carbon emissions are dominated by heating owing to a reliance on fossil fuel boilers [2]. Since these boilers, by definition, release greenhouse gases to produce heat, they cannot be decarbonized without intervention in our existing buildings.

In Europe, the European Union (EU) has set out plans to phase out boilers powered by fossil fuels completely by 2040 [3]. Globally, other heating dominated nations also aim to remove non-renewable heat sources. For instance, the UK plan to stop new installations of gas boilers by 2035 [4] and Canada would need 10% of homes to be heated by heat pumps to meet 2030 climate targets [5]. Replacement of these systems with low-carbon alternatives such as heat pumps presents additional challenges including the sustainable and constant provision of electricity [6]. The electrification of the built environment will only be a carbon-neutral strategy when paired with successful decarbonization of electricity production. This decarbonization will require larger levels of intervention as consumption increases.

Therefore, building fabric retrofit, including insulation and air tightness improvements, remain essential in improving energy efficiency to minimize the increase in electricity consumption resulting from the transition away from fossil fuels. In fact, in Europe, approximately 97% of stock needs upgrading in efficiency to meet 2050 decarbonization targets [7]. With over 200 million buildings in the EU and Switzerland alone [8], this demonstrates the required level of mass intervention. The material choice of this intervention and its embodied impacts has also been shown to be pivotal to whether a country can meet its retrofit carbon budgets, showing the importance of considering emissions over a building’s entire lifespan when employing carbon mitigation strategies [9].

The importance of understanding whole-life carbon emissions becomes evident when comparing retrofit to the alternative of demolition and new construction. This comparison, made for a residential case study, shows very different results depending on whether only an upfront embodied carbon assessment is undertaken or a whole life analysis [10]. The upfront embodied carbon savings of the retrofit compared to the new construction scenario are significantly diminished over the building’s lifespan owing to the worse operational performance of the retrofit case [10]. These findings demonstrate the importance of understanding the balance between operational and embodied carbon when considering future interventions to the stock. A systematic review of existing work also shows that the majority of publications undertake such analysis at the individual building level [11]. There also exists a wide variation in the methodologies adopted [11,12], e.g. which stages of the building lifecycle are assessed [11] and what lifespan/study period is adopted [11]. While demonstrative, these case studies, however, pose a disadvantage. As building characteristics, e.g. fabric efficiency and geometry, are likely to change between individual case studies, it thus remains difficult to draw generalized trends from the work to date.

Both the efficacy of building fabric retrofit and material consumption are related to a building’s geometry, which can be defined as its geometric form. Building form defines the relationship between exposed surface area and the internal volume of a building. Therefore, form provides a proxy for the relative importance of the heat losses through walls, floor and roof and the heat loss through the infiltration of cold outdoor air owing to ventilation losses. Consequently, buildings of the same fabric characteristics can have different energy consumption because higher exposed area compared to the heated internal volume and floor area. In fact, form is shown to impact the energy efficiency of a building [13–16] as well as the embodied carbon of new constructions [15]. From a retrofit perspective, the required insulation levels to achieve a similar energy efficiency level are higher for buildings with a larger volume-to-floor area ratio [17]. Therefore, understanding the impacts of form has applications for whole-life carbon assessments of retrofit as it impacts both operational and embodied carbon of a building. At scale, a national residential stock study shows that prioritizing retrofit of the worst performing buildings assists with meeting governmental carbon reduction targets [18]. As form impacts the energy efficiency of a building [13–16], there are further potential applications to use form to identify and prioritize retrofit where it will make the largest impact on total emissions. However, it is not understood how building form influences which intervention scenario for existing buildings will cause the lowest carbon emissions from both an energy and material consumption standpoint. It is this gap in knowledge we seek to fill.

In this work, we offer a parametric formulation of whole-life emissions as a function of building form and quantify the impact of this on the choice of low-carbon interventions within the built environment. We analytically connect key building form factors with heating energy consumption, total operational and embodied carbon. By making this connection (rather than undertaking singular case study analysis as common in whole-life assessments [11]) a broader understanding of how different building characteristics impact energy consumption and whole-life carbon retrofit decisions is achieved. This allows for rapid assessment and visualization of how future decision-making may change for many different building forms.

The rest of this paper will be organized as follows. We first parametrize building form as a function of form factors and quantify the relationship between building form factors and whole-life carbon intervention decisions. This will then be validated using existing building geometries to demonstrate the potential for these decisions to change within realistic stock types which is followed by concluding remarks.

## 2. Building form and components

Building form can be measured in many ways, with past work typically using a single factor to compare different building designs [13,15–17,19]. For example, one commonly used metric is surface area to volume ratio (S/V), which calculates the ratio of external surface area to internal volume. Buildings with smaller S/V are shown to have lower energy consumption [13,16]. However, this value is not dimensionless and is influenced by building size. Therefore, two buildings of the same form can have different values, which limits the ability to use this factor for direct comparisons between shapes [19]. One measure of compactness has been defined which is the ratio of building surface area (S) to minimum surface area required to enclose the same volume ($S_{\text{min}}$). By creating a scale-independent measure of form this value is not influenced by building size unlike S/V [19].

This measure of compactness uses both aspect ratio (r=W/L), which is the ratio of building width to length and slenderness (k=H/L), which is the ratio of height to length [19]. In multi-storey building design a small aspect ratio, combined with a larger floor area per storey reduces not only heating and cooling energy consumption but the upfront embodied carbon emissions [15].

By defining form as a single parameter such as compactness, S/V, or solely aspect ratio, one cannot distinguish between the potential differences in the thermal performance of different building surfaces. Where this difference is high, which commonly occurs owing to relatively poor performance of glazing compared to other elements, these shape factors do not account for worse performance of certain building forms.

When kept separate, aspect ratio (r) and slenderness (k) account for any differences in performance between fabric elements. This allows for building form to be described for any rectangular building form and distinguish between potential thermal performance variations of different building elements. From these form factors, the analytical connection between form and energy consumption and whole-life carbon can be quantified, allowing for whole-life carbon decision-making for many buildings of different shapes and sizes.

### 2.1. Modelling whole-life carbon as a function of building form

The following sections will use the previously discussed form factors, aspect ratio, r, and slenderness, k, to analytically express both energy consumption [kWh], F, and whole-life carbon emissions [KgCO2e], E, in terms of building form.

First, we describe the developed model, which expresses thermal energy consumption as a function of building form, in a cool-temperate climate. To understand heating energy consumption, we define the heat losses through the building fabric and ventilation in terms of form factors to define the building base temperature [°C], $\theta_{\text{b}}$. The base temperature is defined as the external temperature where no mechanical heating is required and therefore can be compared to the actual external temperature to understand heating requirements. This is a measure commonly calculated when adopting the degree days methodology, an established method that has advantages over extremely simplified methods as it accounts for fluctuation in outdoor temperature [20]. Owing to various simplifications it is seen as having limited accuracy in comparison to more detailed dynamic thermal simulation [20]. However, degree days have been deemed appropriate as a reference point and basis for this body of work, because despite the limitations its ease and speed of implementation make it an ideal choice for very early design decisions where large amounts of detail and inputs, such as exact occupant behaviour, are not likely to be collected [21].

Therefore, by inputting form factors directly into the degree days model we can understand how form influences energy consumption alongside key characteristics of a building, e.g. fabric losses using the U-value ($\text{W/m}{\;}^{-2}\text{K}$), U and ventilation losses using the infiltration rate (${\text{hr}}^{-1}$), N.

Secondly, the parametric contribution of building form to total embodied carbon is demonstrated. Assessing both operational and embodied contributions to carbon emissions allows for total whole-life carbon to be expressed as a function of key building form factors and an understanding of the influence of building form on the lowest whole-life carbon intervention.

We assume this rectangular form throughout the rest of the paper. See of the electronic supplementary material, S3 for a quantification of the impact of this simplification for a wide range of real existing building forms.

## 3. Thermal energy consumption

To understand the impact of retrofit measures, the energy consumption of a building must first be understood. We can describe this by splitting total energy consumption ($\text{k}\;\text{Wh}\;{\text{m}}^{-2}$) into its component parts:


(3.1) $$F_{\text{total}}=F_{\text{heating}}+F_{DHW}+F_{\text{kitchen}}+F_{\text{elec}},$$

where $F_{\text{heating}}$ is the total energy consumption from heating the space, $F_{DHW}$ accounts for total hot water usage by occupants, $F_{\text{kitchen}}$ refers to any fossil fuel usage owing to cooking. $F_{\text{elec}}$ defines the total electricity consumption from occupant behaviour such as equipment and lighting as well as any electrically run mechanical systems such as ventilation and cooling systems.

As this paper focuses on the reduction in fossil fuel usage in a cool-temperate climate [22], the aim is to reduce the total thermal energy consumption in the space. Natural ventilation, with no mechanical cooling, is shown to be a commonly adopted strategy within cool-temperate countries [23,24]. This building services strategy is prevalent in residential [25,26] and non-residential [27] buildings, especially for older buildings [28], which will require highest level of retrofit intervention. These older buildings were built before mechanical ventilation and cooling were typically used in buildings.

Therefore, thermal energy consumption only refers to heating, hot water and kitchen gas usage within the space:


(3.2) $$F_{\text{thermal}}=F_{\text{heating}}+F_{DHW}+F_{\text{kitchen}}.$$

We have made the assumption that $F_{DHW}$ and $F_{\text{kitchen}}$ are independent from building shape and form. Hot water usage has been shown to be dependent on the number of occupants [29] and kitchen gas usage on the number of meals prepared [30]. These values are dependent on occupancy density, O, ($\text{people}\;{\text{m}}^{-2}$) which is independent of form as it is normalized by floor area.

Heating energy consumption, $F_{\text{heating}}$, is the most challenging model to parametrize. The model is reliant on defining the heat loss through building fabric and ventilation, which have a complex relation to building form.

### 3.1. Heating energy consumption

We consider heating energy consumption as a balance between heat lost through a building’s fabric and ventilation and heat gained owing to internal occupant behaviour, solar gains and the additional heat provided by heating systems to ensure a comfortable space.

The rate of heat lost through a building element is dependent on the thermal performance of that element. This can be described using a U-value [$\text{W}\;{\text{m}}^{-2}\text{K}$], U, which is the rate of heat transmittance.

We can describe the total fabric conductance ($\text{W}\;{\text{K}}^{-1}$) as the total area of each element multiplied by their heat transmittance:


(3.3) $$U_{\text{fabric}}^{\prime }=2L^{2}(kr+k)(wU_{\text{wall}}+gU_{\text{window}})+L^{2}r(U_{\text{roof}}+U_{\text{floor}}),$$

where the area has been defined using the length of the building, L, and building form factors slenderness, k and aspect ratio, r. The glazing ratio is g and w=(1−g).

The heat lost through ventilation is dependent on the amount of cold air being exchanged with indoor air. This is owing to necessary ventilation to provide sufficient fresh air levels to occupants and unwanted infiltration owing to leaks in the building fabric. The total ventilation conductance ($\text{W}\;{\text{K}}^{-1}$) is dependent on the total volume flow rate of this air (${\text{m}}^{3}\;\text{h}{\text{r}}^{-1}$), $q_{v}$ and its respective thermal properties:


(3.4) $$U_{\text{ventilation}}^{\prime }=\frac{1}{3600}q_{v}c_{p}\rho ,$$

where $c_{p}$ is the specific heat capacity (${\text{K}}^{\left(-1\right)}:\text{J}({\text{kg}}^{-1})({\text{K}}^{-1})$) and ρ is the density ($\text{k}\text{g}\;{\text{m}}^{-3}$) of air at that temperature. As these properties do not change much between different typical air temperatures a constant value can be assumed. This value is 1/3 [21].

We define the ventilation rate, N, as the total number of complete exchanges of outdoor air within the building per hour, (${\text{hr}}^{-1}$), which allows for the volumetric flow rate of air to be expressed as:


(3.5) $$U_{\text{ventilation}}^{\prime }=\frac{NL^{2}xrH_{\text{fc}}}{3},$$

where x is the number of floors in the building and $H_{\text{fc}}$ is the internal floor to ceiling height (m), which is assumed to be a constant typical value across floors.

The sum of $U_{\text{fabric}}^{\prime }$ and $U_{\text{ventilation}}^{\prime }$ provides the total building heat loss coefficient ($\text{k}\text{W}\;{\text{K}}^{-1}$):


(3.6) $$U^{\prime }=L^{2}\frac{2(kr+k)(wU_{\text{wall}}+gU_{\text{window}})+rU_{\text{roof}}+rU_{\text{floor}}+\frac{NxrH_{\text{fc}}}{3}}{1000},$$

where the values have been divided by 1000 to covert from Watts to kiloWatts as typically heating energy consumption is given in $\text{k}\text{Wh}\;{\text{m}}^{-2}$.

We can use this value to determine the rate of heat lost or gained (kW) from a building’s fabric and ventilation at any given internal required, $\theta_{\text{sp}}$, and external temperature, $\theta_{o}$, (°C):


(3.7) $$Q_{\text{loss}}=U^{\prime }(\theta_{\text{sp}}-\theta_{o}).$$

Occupants, electronic equipment and lighting as well as solar energy through glazing all produce heat within a space. These internal gains allow for heating energy consumption to be lower than the total heat lost through a building’s fabric and ventilation.

We calculate occupancy related internal gains ($\text{kW}\;{\text{m}}^{-2}$), $q_{o}$, by averaging typical occupant, equipment and lighting gains over a 24 h period. See the electronic supplementary material for all occupant data used within the study.

Solar gains through glazing, $q_{\text{s}}$, must also be calculated and averaged over the 24 h period. Given our focus on the relationship between building form and this impact on energy consumption, an average solar gain value, $q_{sol}$, ($\text{kW}\;{\text{m}}^{-2}$) has been adopted. $q_{\text{sol}}$ is calculated as the average solar gain on each wall for a square-plan, south facing building. The glazing ratio is assumed to be constant for each wall. See the electronic supplementary material, S5.4 for sensitivity analysis on this assumption, which shows on average only a very small impact on results. Therefore, we approximate $q_{s}$ as:


(3.8) $$q_{s}=q_{\text{sol}}s_{g}f,$$

where $s_{g}$ is the solar gain factor, which dictates how much heat is transferred through the glazing and f is the typical frame factor of a window.

The adoption of a gains utilization factor, $\eta^{\prime }$, is outlined in BS EN ISO 34790 and is used to help account for the temperature variations during the day within the building and materials. This is calculated using the method outlined in CIBSE Guide TM41:2006 [20] and is dependent on the total internal gains to the space as well the heat loss coefficient:


(3.9) $$\begin{array}{lcr} & Q_{g}=[2L^{2}q_{s}g(kr+k)+L^{2}xrq_{o}]\eta^{\prime }. & \end{array}$$

We can calculate the total corrected internal gains, $Q_{g}$, and use them to correct our internal temperature:


(3.10) $$\theta_{b}=\theta_{\text{sp}}-\frac{q_{g}}{Z},$$

where $Z=U^{\prime }/L^{2}$ and $q_{g}=Q_{g}/L^{2}$ and shows that $L^{2}$ is cancelled out of this equation. $\theta_{b}$ is the internal base temperature, defined as the external temperature where no mechanical heating is required.

We substitute $\theta_{b}$ in equation (3.7) to estimate heat lost from the building at any given time of the year. Taking the sum over the entire heating season when the building is occupied and heated and dividing by the floor area, $A=L^{2}xr$, we can estimate the total energy consumption intensity over the year ($\text{kWh}\;{\text{m}}^{-2}$) as:


(3.11) $$F_{\text{heating}}=\sum_{m}^{}\frac{24N_{m}Z}{xr\eta }(\theta_{b}-\theta_{\text{om}}),$$

where $N_{m}$ is the total days in the month, η is the overall efficiency of the heating system and $\theta_{\text{om}}$ is the average monthly outdoor temperature.

As a constant $\theta_{\text{om}}$ is used, we include a correction factor proposed by Hitchin [20] to estimate the impact of varying temperatures throughout the month:


(3.12) $$F_{\text{heating}}=\sum_{m}^{}\frac{24N_{m}Z}{xr\eta }\frac{\left(\theta_{b}-\theta_{\text{om}}\right)}{1-e^{-\frac{2.5}{\sigma_{\theta }}(\theta_{b}-\theta_{\text{om}})}},$$

where $2.5/\sigma_{\theta }$ is a location specific constant to account for the standard deviation in temperature throughout the month [20].

Equation (3.10) does not account for the impact of occupied and unoccupied hours throughout a 24 h period. Building heating systems are, however, switched off for a period when the building is not occupied. A building will store some heat for a period after the heating system is switched off. Further, building heating systems are typically switched on before required occupancy to allow for preheating the space. Therefore, average internal temperature differs over 24 h from either the required internal temperature, $\theta_{sp}$, or the external temperature. To account for this and adjust $\theta_{\text{sp}}$ accordingly, we take account of the thermal properties of the building to understand how much heat could be stored within the fabric. τ, the thermal time constant (hr) is used to understand the response time of the building to temperature changes, where different thermal mass and thermal performance will impact this:


(3.13) $$\tau =\frac{2(kr+k)(wc_{\text{wall}}+gc_{\text{window}})+xrc_{\text{ceiling}}+xrc_{\text{floor}}}{3600Z}.$$

The total thermal capacitance, c, ($\text{kJ}\;{\text{K}}^{-1}.{\text{m}}^{-2}$) of each exposed building element, is defined as density multiplied by specific heat capacity multiplied by the exposed thickness of the material, which is then multiplied by the exposed area of the element.

We also need to estimate the size of the plant within the building as this is used to understand the preheat time before occupancy begins. We simplify plant size calculations, by not accounting for the impact of different types of operation, as the degree days model is shown to be relatively insensitive to plant size [20]. Therefore, we can define plant size (kW), $Q_{p}$, as a function of the heat lost in the space during the coldest period of the year:


(3.14) $$Q_{p}=\frac{\beta (\theta_{\text{sp}}-\theta_{\text{winter}})}{\delta }L^{2}Z,$$

where δ accounts for heat losses owing to distribution within the space and β is a multiplier to oversize the building plant as is commonly undertaken in practice [27]. $\theta_{\text{winter}}$ is the minimum design temperature for a cold temperate climate.

We estimate the preheat period, Ψ, and the switch off period, Ω, by inputting the equations (3.6), (3.13) and (3.14) into the methodology outlined in degree days TM41:2006 [20] and rearranging to cancel out all geometric parameters expect for τ:


(3.15) $$\Psi =-ln[\frac{Y^{2}-2Y\delta \theta +\delta \theta^{2}}{Y^{2}-Y\delta \theta }+(\frac{\delta \theta Y-\delta \theta^{2}}{Y^{2}-Y\delta \theta })e^{\frac{-t_{u}}{\tau }}],$$

(3.16) $$\Omega =\frac{t_{u}}{\tau }+ln[\frac{Y^{2}-2Y\delta \theta +\delta \theta^{2}}{Y^{2}-Y\delta \theta }+(\frac{d\theta Y-\delta \theta^{2}}{Y^{2}-Y\delta \theta })e^{\frac{-t_{u}}{\tau }}],$$

where $\delta \theta =\theta_{\text{sp}}-\theta_{o}$, $Y=Q_{p}/L^{2}$ and $t_{u}$ is unoccupied hours in the space. These values allow us to adjust our indoor temperature, to account for differences over the 24 h period [20]:


(3.17) $$\bar{\theta_{i}}=\frac{\theta_{o}t_{u}}{24}+\frac{\tau (\theta_{\text{sp}}-\theta_{o})}{24}[e^{\Psi }-e^{-\Omega }]+\frac{\tau Y}{24}[1+\Psi -e^{\Psi }]+\frac{\theta_{\text{sp}}t_{o}}{24},$$

where $t_{o}$ is the occupied hours. This value shows that $L^{2}$ is completely cancelled out leaving the only geometric parameters within the equation—slenderness, k, aspect ratio, r, and number of floors, x.

This can be inputted into equation (3.12) to provide an estimate of heating energy consumption for a building of rectangular form.

Within this model, there are key parameters, which will be simplified to constants, as their majority influence is from either building efficiency or the outdoor conditions rather than the geometry of the building. These are the denominator ($D=1-e^{2.5/\sigma_{\theta }(\theta_{b}-\theta_{\text{om}})}$), $\eta^{\prime }$, Ψ and Ω. The demonstration of this for different possible performance levels of a building is demonstrated in the electronic supplementary material, S4.

Constant values for D, Ψ, Ω and $\eta^{\prime }$ have therefore been estimated, to still account for the impact of these factors. Each value is calculated to be applicable to a wide range of building forms. These values are estimated depending on the building performance and typology but use typical geometry to create a constant figure. This typical geometry is based on the typology of the building, using the average floor area of that typology and assuming a simple square-plan building. As the building energy efficiency improves, e.g. reductions in the heat loss coefficient,the impacts of solar and internal gains as well as internal mass become more important. This implies the impact of assuming constant values is more likely to impact model accuracy.

We make an assumption that the denominator, D, is equal to one. This assumption is most appropriate in cold temperatures, however the value of D does decrease as temperatures increase (for corroborating data, the electronic supplementary material, S$). Warmer periods within the heating season have lower energy consumption and it is likely that there will be a slight underestimation of the total annual energy consumption.

### 3.2. Final analytical expression for thermal energy consumption

Having taken D, $\eta^{\prime }$, Ψ and Ω as constants, the only remaining variables in equations (3.12) and (3.17) that are influenced by building form are the values of Z and τ, which are both divided by aspect ratio and number of floors as shown in equation (3.12).

We can separate these values into three respective parts. The first being all the heat losses, thermal mass and heat gains which are influenced by the form of the building walls and glazing (k/x+kr/x):


(3.18) $$\begin{array}{r} C_{1}=\frac{N_{m}}{\eta D}[(\theta_{o}t_{u}+\theta_{sp}t_{o}-24\theta_{o})(\frac{2wU_{\text{wall}}+2gU_{\text{window}}}{1000})]-\frac{48gN_{m}q_{s}\eta^{\prime }}{\eta D}+\\ {}[\frac{N_{m}\delta \theta }{3600\eta D}(e^{\Psi }-e^{-\Omega })+\frac{N_{m}Y}{3600\eta D}(1+\Psi -e^{\Psi })](2wc_{\text{wall}}+2gc_{\text{window}})). \end{array}$$

The second, aggregates the influence of the heat losses through the roof and ground floor (1/x):


(3.19) $$C_{2}=\frac{N_{m}}{\eta D}[(\theta_{o}t_{u}+\theta_{\text{sp}}t_{o}-24\theta_{o})(\frac{U_{\text{roof}}+U_{\text{floor}}}{1000})].$$

Finally, the third provides an indication of the effects of internal gains and thermal mass of the roof and floors, which are not influenced by building form so are therefore constant:


(3.20) $$\begin{array}{rl} C_{3} & =\frac{N_{m}}{\eta D}[(\theta_{o}t_{u}+\theta_{\text{sp}}t_{o}-24\theta_{o})(\frac{NH_{\text{fc}}}{3000})]-\frac{24N_{m}\eta^{\prime }q_{o}}{\eta D}+\\ & [\frac{N_{m}\delta \theta }{3600\eta D}(e^{\Psi }-e^{-\Omega })+\frac{N_{m}Y}{3600\eta D}(1+\Psi -e^{\Psi })](c_{\text{roof}}+c_{\text{floor}}). \end{array}$$

Together, these allow us to express heating energy consumption as:


(3.21) $$F_{\text{heating}}=C_{1}\frac{k}{x}+C_{1}\frac{k}{xr}+\frac{C_{2}}{x}+C_{3}.$$

As previously stated, we can also bundle the total hot water usage and kitchen gas usage as a function of the occupancy density ($\text{people}\;{\text{m}}^{-2}$), O. These are by definition independent of form when energy consumption is normalized per floor area. This allows us to express the values of $F_{DHW}$ and $F_{\text{kitchen}}$ as a constant:


(3.22) $$C_{4}=\mu O\kappa N_{t}+\sum_{m}^{}\frac{4.18LN_{m}\delta \theta_{DHW}}{3600\eta },$$

where $N_{t}$ is the total number of occupied days, μ is a meal to total occupancy ratio, κ is typical kitchen gas usage ($\text{kWh}\;{\text{meal}}^{-1}$). L is typical hot water usage ($\text{L}\;{\text{m}}^{-2}$) and $\delta \theta_{DHW}$ is the monthly typical temperature difference of incoming cold water and the hot water system [31]: 4.18 refers to the specific heat capacity of water and 3600 converts results from kJ to kWh.

This allows us to express total building thermal energy usage as:


(3.23) $$F_{\text{thermal}}=C_{1}\frac{k}{x}+C_{1}\frac{k}{xr}+\frac{C_{2}}{x}+C_{3}+C_{4}.$$

### 3.3. Fit of the analytical expression to degree days

Equation (3.23) shows the relationship between k, r and x and energy consumption. As we make simplifications in the calculation of $C_{1}$, $C_{2}$, $C_{3}$ and $C_{4}$, we therefore investigate the appropriateness of the form of analytical expression itself e.g. $F=a\frac{k}{x}+a\frac{k}{xr}+\frac{b}{x}+c$ . A comparison is now made between the developed expression and the degree days model as discussed in §2.1.

This comparison is undertaken by fitting the form of $a\frac{k}{x}+a\frac{k}{xr}+\frac{b}{x}+c$ directly to degree days modelling results for a sample of buildings—all UK educational polygons modelled as rectangular forms. Degree days modelling of thermal energy consumption is undertaken for all polygons for different levels of thermal fabric efficiency using methods outlined in CIBSE TM41 [20].

Results of this process model energy consumption ($\text{kWh}\;{\text{m}}^{-2}$), y, using an already established model [20]. Slenderness and aspect ratio values are estimated for all buildings, and nonlinear least squares is used to fit the form of $a\frac{k}{x}+a\frac{k}{xr}+\frac{b}{x}+c$ to the data. Therefore, we now have values of a, b and c that best fit degree days modelling results, y. This allows for energy consumption (kWh) to be calculated once more from the analytical expression as $y_{0}$.

By comparing the degree days model, y to the results of the analytical expression, $y_{0}$ we can understand how well the form of this expression fits established energy modelling results.

An extremely close correlation between the two results ($y,y_{0}$) is found and shown in table 1. In fact, the average percentage error is 0% with a very low standard deviation of less than 1% for both building performance levels. This shows that the analytical expression works well for a variety of rectangular building forms as well as different fabric and ventilation performance levels. For further data associated with these results please see the electronic supplementary material, S4.1.

**Table 1.** Comparisons between degree days modelling results, y and $y_{0}$ . ($y_{0}$ has been estimated by fitting the form of $a\frac{k}{x}+a\frac{k}{xr}+\frac{b}{x}+c$ to degree days modelling results, y . Table shows the line of best fit between y and $y_{0}$ as well as the correlation coefficient $R^{2}$ , the average percentage error $\bar{\text{error}}$ and the standard deviation of that percentage error, $\pm \sigma_{\text{error}}$ . Two extremes of building performance are modelled—historic which assumes a completely uninsulated building and EnerPHit which is a retrofit standard dictating extreme fabric and ventilation standards.)

thermal performance	results to examine the extent to which the expression in the form of $a\frac{k}{x}+a\frac{k}{xr}+\frac{b}{x}+c$,fits to the degree days energy model			
	equation of best fit between y and $y_{0}$	$R^{2}$	$\bar{\text{error}}$ (%)	$\pm \sigma_{\text{error}}$ (%)
historic— very poor thermal performance	$y_{0}=0.99y+1.0$	0.99	0	0.88
EnerPHit —very high thermal performance	$y_{0}=0.99y+0.4$	0.99	0	0.03

This section shows that the form of the analytical expression can be used to describe energy consumption of different performance levels to the same extent as the degree days model when rectangular form is assumed. Previous work has used degree days modelling, to understand predicted energy consumption compared with metered energy data for a non-residential cool-temperate case study [32]. In this case study, it is shown that a degree days model, alongside simplified input data, e.g. occupancy profiles and weather data, is sufficient to understand the energy consumption of the building [32]. The connection between degree days and metered energy consumption for residential properties is also demonstrated, though using measured rather than modelled average internal temperature [33].

### 3.4. The connection between thermal energy and building form

Equation (3.23) demonstrates that energy consumption increases as slenderness increases and aspect ratio decreases. This is logical as those buildings with a larger slenderness will have a larger wall area to heat the same floor area and those with a smaller aspect ratio will have a larger exposed perimeter. The form of this equation also demonstrates that differences in the ratio of wall and floor area will impact energy consumption due to the differences in performance of these elements.

**Figure 1.**
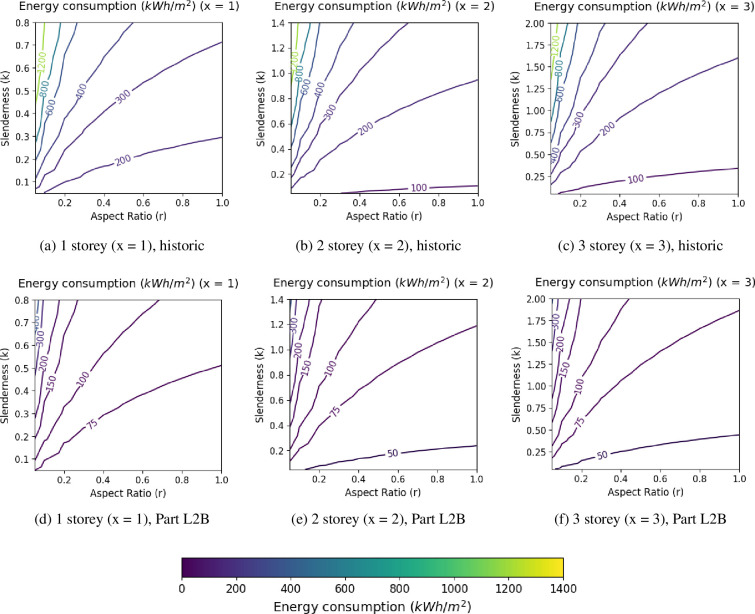
Contour plots to show the distribution of thermal energy consumption ($\text{kWh}\;{\text{m}}^{-2}$) in relation to slenderness, k, and aspect ratio, r as a function of the number of storeys, x. Fabric performance levels have been modelled as a typical historic building and at Part L2B levels—Refer to the electronic supplementary material for modelling data. For each value of x the typical realistic ranges in slenderness were found from analysis of existing buildings.

The analytical expression, equation (3.23), is demonstrated in figure 1, for two different building performance levels. UK weather patterns have been modelled as an example cool-temperate climate and a primary school typology is modelled as within the UK, these buildings are typically naturally ventilated with no mechanical cooling [34]. Figure 1a–c show the analytical expression for 1, 2 and 3 storey buildings modelled as a typical pre 1919 (historical) building, with uninsulated walls, roof and floor, single glazing and a gas boiler. Figures 1d–f model Part L2B performance level. This is a level of retrofit outlined by UK building regulations for non-residential buildings. Therefore, this standard is assumed to be a typical and achievable level of improvement to fabric and ventilation efficiencies. Figures 1–f only accounts for thermal energy consumption, and it is assumed the building remains naturally ventilated.

These results demonstrate that energy saving potential changes for different building forms. Part L2B buildings have a smaller range in relative energy consumption compared to historic. Therefore, the total energy consumption of Part L2B buildings is less influenced by building form. This implies that retrofit of those historic buildings with an inefficient form will provide larger energy savings than those with efficient forms. In this piece of work, the term *efficient form* refers to those buildings with a low slenderness combined with a high aspect ratio, therefore having lower energy consumption compared to other forms built to the same fabric standards. As evidence shows a need for immediate carbon emission reductions [35], from an at scale perspective, prioritizing retrofitting the most inefficient forms first could be beneficial to meeting carbon targets. Analysis of the Irish residential sector shows that prioritization of retrofit for the worst performance homes will be required to meet national reduction targets [18], showing that an understanding of the influence of form could be highly useful in future decision-making.

## 4. Whole-life carbon decision-making

We can now use the parametrized thermal energy consumption and combine this with predicted embodied emissions from different potential intervention measures to understand the influence of building form on retrofit decision making.

Two possible interventions are retrofit of the existing building to a higher standard or demolition and replacement with a more efficient construction. These would both reduce the values of our surface constants, $C_{1}-C_{4}$, compared to the base case. The embodied carbon input of demolition and construction would clearly be high in comparison, but there are several factors, such as building form, within existing buildings that can make it harder to achieve the same energy consumption as a new build [17]. There are conflicting results within existing studies, some showing retrofit have lower environmental impacts [36–46] and others having scenarios where new construction is preferable [47–54] in whole life emissions.

As new construction is not confined by the existing form of the building, this provides an interesting whole-life carbon comparison between different new construction standards and retrofit scenarios. Therefore, we aim to quantify the parametric connection between operational carbon, embodied carbon and building form and compare different intervention strategies from a whole-life carbon perspective.

Equation (3.23) demonstrates the relationship between thermal energy consumption and slenderness, aspect ratio and number of storeys. We can also use this form to describe total whole-life carbon emissions as the operational emissions(${\text{kgCO}}_{2}\text{e}\;{\text{m}}^{-2}$), $E_{\text{oc}}$, are a function of the total energy consumption. Embodied carbon (${\text{kgCO}}_{2}\text{e}\;{\text{m}}^{-2}$), $E_{\text{ec}}$, is dependant of the form of building walls, roof and floor which we have already described within equation (3.23).

We estimate yearly operational carbon as the yearly energy consumption multiplied by the respective operational carbon factor, o, of the fuel, f, used:


(4.1)Eoc=ofFthermal+ofFelectricity(4.2)=of(C1kx+C1kxr+C2x+C3+C4)+Cec,

where $F_{\text{electricity}}$ (${\text{kgCO}}_{2}\text{e}\;{\text{m}}^{-2}$) is a constant operational energy benchmark for electricity consumption from occupant behaviour, such as lighting and equipment. As this paper focuses on the reduction of thermal energy consumption, electricity consumption is assumed to be constant depending on the typology of the building. Therefore, we assume that lighting consumption is not affected by building form. The electronic supplementary material, S5.2 describes how we estimated this value using historic metered energy consumption.

Total $C_{\text{oc}}$ is then calculated by summing over the appropriate lifespan of the building.

We can now calculate embodied carbon of each retrofit scenario. Those which impact wall and glazing area include the addition of wall insulation and glazing replacement. Also included in this is the replacement of existing boilers. This is because the size of the replacement boiler can be estimated as a function of the building heat loss coefficient (see equation (3.14)), which is dependant on the building form [21]. The total influence of the form of wall area on total embodied carbon can be understood as:


(4.3) $$C_{\text{ec1}}=(e_{\text{wall}}w)+(e_{\text{window}}g)+\frac{\left(2e_{\text{boiler}}Y)(wU_{\text{wall}}+gU_{\text{window}}\right)}{1000},$$

where $e_{\text{wall}}$ (${\text{KgCo}}_{2}\text{e}\;{\text{m}}^{-2}$) is the embodied carbon of roof insulation, $e_{\text{window}}$ the embodied carbon of replacing existing windows (${\text{kgCO}}_{2}\text{e}\;{\text{m}}^{-2}$) and $e_{\text{boiler}}$ the embodied carbon of replacing the heating system [${\text{kgCO}}_{2}\text{e}\;{\text{KW}}^{-1}$].

Insulation of the floor and roof can quantified alongside the influence of heat losses through the roof and floor on total replacement boiler size:


(4.4) $$C_{\text{ec2}}=(e_{\text{floor}})+(e_{\text{roof}})+\frac{\left(e_{\text{boiler}}Y)(U_{\text{floor}}+U_{\text{roof}}\right)}{1000},$$

where $e_{\text{floor}}$ and $e_{\text{roof}}$ are the embodied carbon of insulation measures for each element (${\text{kgCO}}_{2}\text{e}\;{\text{m}}^{-2}$).

Embodied carbon values which are independent of building form such as hot water boiler installation as well as the influence of ventilation losses on the replacement boiler size can now be quantified:


(4.5) $$C_{\text{ec3}}=\frac{\left(e_{\text{boiler}}YNH_{fc}\right)}{3000}+(e_{\text{boiler}}Q_{DHW}),$$

where the size of the DHW system, $Q_{DHW}$, is estimated by calculating the size required to provide sufficient hot water to the occupant based on occupancy patterns.

We can sum up the respective operational carbon and embodied carbon for each element of building form the express total whole-life carbon (${\text{KgCO}}_{2}\text{e}\;{\text{m}}^{-2}$) as:


EWLC=(Coc1+Cec1)kx+(Coc1+Cec1)kxr(4.6)+(Coc2+Cec2)x+(Coc3+Cec3+Coc4+Cec).

The whole-life carbon emissions of different carbon mitigation scenarios can then be superimposed over each other where the lowest carbon option is given for different values of slenderness and aspect ratio. This provides an early stage decision-making for the demolition versus retrofit of existing buildings.

Those scenarios with more efficient building fabric and systems often require larger input of materials to achieve this. Therefore, though operational carbon, $C_{\text{oc}}$, may be decreasing, embodied carbon would be increasing, $C_{\text{ec}}$ making the balance between this and the building form important to finding the lowest carbon intervention measures for our stock.

### 4.1. The connection between retrofit decisions and building form

Four typical intervention scenarios are modelled, assuming the existing building is of a historic standard. These will be modelled for one-storey buildings as this is typical of primary schools [55], which is the focus of this study. The retrofit scenario has been designed to try and reflect what may be typically undertaken, using existing standards in industry. PartL2B refers to UK building regulations [56], and provides limiting U-values for standard of refurbishment:

(i) **baseline**: no intervention and embodied carbon is zero;
(ii) **Part L2B retrofit**: upgrading of thermal fabric to Part L2B standards as well as the replacement of the old existing boiler with an air-to-water heat pump. Building emitters are also replaced owing to evidence that lower supply temperatures require larger surface area [57];
(iii) **current aspirational new construction target**: a new building modelled to meet the (Royal Institution of British Architects (RIBA) 2030) climate challenge [58], 2030 standards. This will be modelled as running on only electricity; and
(iv) **predicted future new construction target**: a new building modelled to the most efficient available embodied carbon target. This was developed by the low energy transformation initiative (LETI) and is referred to as LETI A+whole-life carbon target [59]. This target has an extremely low embodied carbon value, not likely achievable at scale, at this time. This will be modelled as running on only electricity.

Both new construction scenarios will be assumed to be independent of form, using typical existing benchmarks (${\text{KgCO}}_{2}\text{e}\;{\text{m}}^{-2}$) taken from industry [58,59].

In the case of mechanical, electrical, and plumbing replacement, Part L2B only requires the upgrade of systems to the same level as they were before. For this reason these scenarios remain a naturally ventilated space. As historical buildings are being modelled the retrofit scenarios are chosen to be sensitive to this, including internal rather than external insulation and the roof assumed to be pitched [60], which makes loft insulation the retrofit measure for this element.

Embodied carbon data has been collected using cradle-to-grave boundary conditions, which include production, transportation, wasted materials, maintenance and repair as well as the demolition and disposal emissions. This includes the replacement of any elements whose lifespan falls below the building lifespan. An additional benchmark value of $35\;{\text{kgCO}}_{2}\text{e}\;{\text{m}}^{-2}$ will also be included to account for the demolition of the existing building at the end of its lifespan or when it is modelled to be replaced [61].

The results have been quantified over a chosen 30 year lifespan, as this is the period where current net zero targets are meant to be met, by 2050. Three different glazing ratios have been modelled —15% to give a minimal value, 25% which is typical of educational stock [62] and 45% to give an upper boundary.

The decarbonization of the electric grid was modelled using the UK national grids ‘Future energy scenario’ called ‘falling short’ [63], with carbon capture excluded.

**Figure 2.**
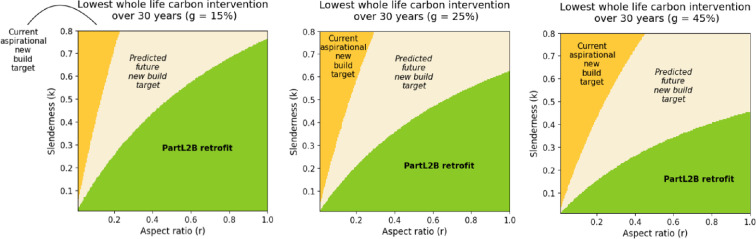
Lowest carbon intervention measure comparing Part L2B (UK building regulation) retrofit, and two new build scenarios for different values of slenderness, k, and aspect ratio, r, and three different glazing ratios, g. Electricity decarbonization has been modelled using the UK national grid `falling short' scenario excluding carbon capture and storage. Different colours describe where each intervention option provides the lowest carbon scenario over the 30-year study period for different values of slenderness and aspect ratio. (a) one storey (*x* = 1), 15%~glazed. (b) one storey (*x* = 1), 25%~glazed. (c) one storey (*x* = 1), 45%~glazed.

Figure 2 demonstrates the impact of building form on the lowest whole-life carbon intervention when comparing retention, retrofit and demolition with new construction. Assessing all figure 2a–c shows that the baseline gas model is never considered optimal which is owing to its inability to decarbonize over the 30 year period. This finding occurs in spite of lower embodied carbon emissions for the baseline scenario.

Figure 2b provides results for a typical primary school [62], with a glazing ratio of 25%. Figure 2b shows that more efficient forms with a low slenderness and high aspect ratio, require less severe intervention to still achieve low carbon emissions. The importance of embodied carbon modelling is shown as the currently aspirational new construction scenario only has lower carbon emissions in a small proportion of highly inefficient building forms, despite achieving very high operational efficiency. The importance of material emissions is further emphasized when you consider that the current aspirational target modelled achieves far better efficiency, with an embodied carbon of $540\;{\text{KgCO}}_{2}\text{e}\;{\text{m}}^{-2}$, than standard practice as the RIBA states the current business as usual embodied carbon benchmark to be $1000\;{\text{KgCO}}_{2}\text{e}\;{\text{m}}^{-2}$ [58].

The predicted future new construction standard, with a total embodied carbon of just $260\;{\text{KgCO}}_{2}\text{e}\;{\text{m}}^{-2}$ [59], takes up a considerably larger area of the heat map. This implies that where very low embodied carbon is achieved, building form becomes more pivotal in the decision-making process.

Figure 2c shows that as the glazing ratio is increased there is a larger range of building forms where new construction is preferable. This is because higher glazing ratios have larger heating energy consumption, owing to the less efficient *U*-values of glass, and so more intrusive intervention achieves the lowest carbon emissions over a wider range of building forms. Similarly, as in figure 2a, reducing the glazing ratio reduces the range of building forms where new construction is preferable. The impact of reducing the glazing ratio to 15% is considerable with an additional 10% of the total graph area where Part L2B is now the lowest carbon intervention compared to the 25% glazed model.

Where past work typically focuses on singular case studies [11], the model developed here allows for a graphical presentation and a broad understanding of the patterns and trends in energy consumption as well as the implications for whole-life carbon decision-making for existing buildings.

## 5. Application to existing buildings

To demonstrate the potential for retrofit decisions to be influenced by form in the existing building stock, a set of real buildings has been modelled.

The focus of this case study is on existing non-residential buildings, as non-residential buildings are more likely to vary with respect to their geometry. Past publications show a higher variation in building size [64] and building depth [65] compared to residential buildings. Non-residential buildings are also shown to have higher replacement rates [66], making the use of whole-life carbon modelling more pressing in early design stages to reduce potentially excessive carbon emissions from demolition and rebuild.

The entirety of UK educational building polygon data was collected from Verisk UKBuildings [67] providing a wide range of building ages, heights and forms. Educational stock was chosen as these polygons provide a wide range of stand-alone buildings, which can be explored with a reduced risk of different construction typologies and building uses being merged together; see the electronic supplementary material for the data collection process. The number of floors (x) was estimated as the value where the floor-to-ceiling heights fit within or was as close as possible to the typical range of 2.4mto4.5m [27]. This shows that the building models used within this section are a theoretical estimate of the actual building. By using realistic polygons this should allow for a more practical assessment of the model. Post-processing leaves a total of 76 231 polygons.

Assuming rectangular form for all these buildings, slenderness, k, and aspect ratio, r, can be calculated by finding the width, length and height of each polygon, where k=H/L and r=W/L. The following section will undertake modelling assuming that all buildings are very old and inefficient, built to historical UK standards. However, the model can be applied to naturally ventilated buildings of many different types as long as the thermal fabric and occupancy patterns of the building are understood, in order to be able to calculate values of C using equations (3.18)−(4.5).

### 5.1. Intervention decisions

To understand the lowest carbon retrofit decisions for our actual stock, the developed model was run for existing buildings using the scenarios outlined in §4. From the wider educational building stock, predicted one-storey buildings were isolated as is typical of primary schools [55] leading to a total of 15 193 building forms. These are all modelled as a worst-case historical building.

Alongside ‘falling short’ grid predictions, another grid scenario was modelled—‘leading the way’ which assumes the UK national grid’s best-case scenario for grid decarbonization.

**Figure 3.**
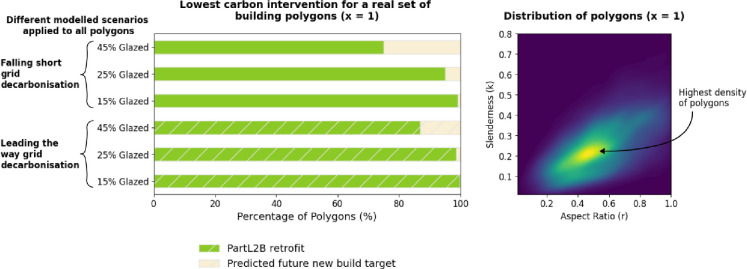
(a) Bar charts to show the distribution of lowest carbon scenarios for different electricity decarbonization rates and glazing ratios when applied to one storey UK educational polygons. Hatching is used to identify leading the way grid decarbonization. No polygon would have the currently aspirational new build target as the lowest carbon solution. (b) Kernel density estimate plot demonstrates the distribution of slenderness and aspect ratio values for one storey UK educational polygons within the sample. This distribution is plotted in its entirety for different glazing ratios and future electric grid scenarios.

Figure 3b shows the distribution of slenderness and aspect ratio for the existing building polygons. This shows that the majority of realistic building forms fit within the right-hand, lower corner of the graph where the differences between energy consumption are less extreme than those with a very high slenderness and low aspect ratio. Figure 3a shows, even with a glazing ratio of 45%, no existing building forms within the sample (where *x* = 1) currently have aspirational new construction as the lowest carbon option.

However, there are differences in the modelled results when comparing future new build standards and Part L2B retrofit. Figure 3a shows that at higher glazing ratios, a proportion of forms would achieve lower carbon emissions if future new build targets were achieved. In fact, a whole-life carbon of $450-500\;{\text{kgCO}}_{2}\text{e}\;{\text{m}}^{-2}$ (stages A1–A5,B1–B5 and C1–C4) for new construction, depending on the operational efficiency, would result in lower carbon emissions for the most inefficient building form within the sample. For context, existing whole-life carbon guidance developed by LETI [59] stipulates a design target in 2030 of less than $400\;{\text{kgCO}}_{2}\text{e}\;{\text{m}}^{-2}$. Therefore, if lower embodied carbon construction becomes feasible in the future, the comparison between new construction and building retention will be more significantly influenced by building form.

The results for one-storey buildings are reflected in the rest of the stock when it comes to the prospect of demolition and replacement. No existing building within the sample has new construction as the lowest carbon scenario, showing that this combination of form factors, with a high slenderness and a low aspect ratio, are potentially unrealistic within the stock. Figure 3a also shows that if the electric grid is able to be decarbonized more successfully, the high embodied carbon of new construction becomes more significant.

It should be noted that these results use specific values for embodied carbon and retrofit performance, of which there is a range of data available. Also, the form of equation (4.6) implies that if we were to find a retrofit scenario that minimizes all values of the constants within the analytical expression, (*C*_1_, *C*_2_ and *C*_3_), we could find the optimal retrofit scenario independent of building form. This is, therefore, a limitation that is constrained to future work, as this may impact the demolition versus refurbishment results. As new construction is not determined by the existing building’s form, the comparison between demolition and refurbishment will always be influenced by form. Therefore, the developed parametric model provides a highly useful tool for comparison.

## 6. Limitations and scope for future work

Although we have shown in §3.3 that the form of the expression equation (3.23) does remain in line with the full implementation of the CIBSE degree days model, we should note limitations to our assumptions when expressing D, $\eta^{\prime }$, Ψ and Ω as constants.

**Figure 4.**
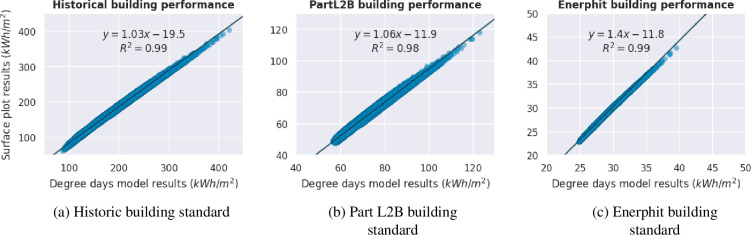
Scatter graph to showing the correlation between the degree days model and the developed analytical expression for total thermal energy consumption for the UK educational stocks existing building forms. Different standards of building efficiency, ranging from historic to EnerPHit, are demonstrated.

We compare our analytical expression and calculations of $C_{1}-C_{4}$ for thermal energy consumption to using full degree days energy modelling. Figure 4a*–*c shows both degree days modelling and the analytical expression have been run for the entire sample of polygons using historic, Part L2B and EnerPHit performance levels, respectively. In comparison to Part L2B, which has been previously investigated, EnerPHit is a level of retrofit equivalent to Passivhaus, that requires a more efficient fabric, strict airtightness levels and specific building systems including mechanical ventilation and heat recovery [68].

Figure 4a, showing the comparison between models for historical buildings, demonstrates a close correlation between the degree days model and the analytical expression. Compared to the degree days model, the developed analytical expression is shown in figure 4a to underestimate energy consumption which, as previously mentioned, is owing to assumption that D≈1. Figure 4b shows that results are very similar for Part L2B performance standards.

The comparison between all figure 4a–c demonstrates that as building performance improves to higher energy efficiency standards the gradient of the graph moves away from y=x. As a building’s performance improves, the relative impacts of the internal gains factor ($\eta^{\prime }$), and thermal time constant, which inform Ψ and Ω, should increase, while being kept as constants here. Therefore, as thermal energy consumption increases, i.e. an increase in slenderness and decrease in aspect ratio, the analytical expression overestimates energy consumption in comparison to full degree days modelling. Figure 4c shows that at extremely high building efficiency levels such as EnerPHit, the gradient of the graph worsens considerably to 1.4 and highlights a limitation of currently developed methods, which is constrained to future work.

For the real set of UK educational buildings this overall error is low with an average percentage error between the analytical expression of ±10%, ±11% and ±3% for historic, Part L2B and EnerPHit buildings, respectively. The assumption of rectangular form has also been further investigated in the electronic supplementary material and shown to cause a 1–3% increase in average error, while not impacting the majority of results which already have rectangular form.

Future work may expand the study boundaries beyond the four scenarios assessed in this publication. Exploring the whole life impacts of a wider set of materials, retrofit measures and grid decarbonization scenarios would allow for a broader understanding of the influence on form when making key future intervention decisions. The system boundaries of this model are also limited to only naturally ventilated buildings, and therefore specific climates where this building system type is commonly found [25–28].

Further, the demolition versus refurbishment comparisons are currently fixed at a lifespan of 30 years. There is evidence that an existing building may not be as structurally durable as an equivalent new construction [51], which demonstrates the limitation of assuming a fixed lifespan. Therefore, future work which accounts for these potential differences, alongside whole-life carbon comparisons would provide a more comprehensive comparison between the options of replacement or retrofit.

## 7. Conclusions

We have shown the relationship between the external surface area and internal volume impacts the energy efficiency of our existing buildings, even if they have the same fabric and ventilation performance. This, in turn, impacts intervention decisions for our existing buildings, specifically the comparison between retrofit and demolition and replacement with new construction. Higher levels of intervention are required for more energy inefficient building forms, with higher slenderness and lower aspect ratio values. The energy saved from retrofit is also larger for more inefficient forms, which when retrofit needs to be applied at scale [7], is an important factor for prioritization of certain forms to ensure maximum, immediate carbon reductions.

New construction is unlikely to provide the lowest carbon option from a carbon perspective for existing buildings using current [58] embodied performance targets of $540\;{\text{kgCO}}_{2}\text{e}\;{\text{m}}^{-2}$. It should also be noted that this is still an aspirational, low embodied carbon, target with current a business as usual benchmark stated to be $1000\;{\text{KgCO}}_{2}\text{e}\;{\text{m}}^{-2}$ [58].

Though there are many different reasons for demolition such as structural and component lifespans, aesthetics and high land value, these results highlight the importance of consideration of whole-life carbon emissions within this decision. Also, this demonstrates a need for reduced embodied carbon emissions in the construction sector if demolition and replacement is indeed required at scale owing to our existing buildings reaching the end of their structural lifespan. As new construction is not constrained by the existing form of the building this work provides a highly useful tool for comparison of typical retrofit scenarios against new construction. Further work could use the method developed here to understand whether retention of buildings with shorter lifespans than new constructions would still be worth retention when considering these emissions.

By adopting shape factors that can define the ratio between wall, roof and floor area this model will allow for quick assessment of whole-life carbon emissions without the requirement for detailed modelling. If building standards improve and the embodied carbon of new construction reduces, building form will become more pivotal within this decision. Therefore, this work provides an extremely time-efficient graphical tool for early stage decision-making and the identification of solutions at scale for planning purposes.

## Data Availability

All data used for this article of work is available through the OS Digimaps platform. Supplementary material is available online [69].
